# Exploring Sexual Dimorphism in the Intestinal Microbiota of the Yellow Drum (*Nibea albiflora*, Sciaenidae)

**DOI:** 10.3389/fmicb.2021.808285

**Published:** 2022-01-05

**Authors:** Haidong Li, Lei Lu, Ruiyi Chen, Shanshan Li, Dongdong Xu

**Affiliations:** ^1^School of Fishery, Zhejiang Ocean University, Zhoushan, China; ^2^Key Lab of Mariculture and Enhancement of Zhejiang Province, Zhejiang Marine Fisheries Research Institute, Zhoushan, China

**Keywords:** sex differences, sexual dimorphism, intestinal microbiota, *Nibea albiflora*, sciaenidae

## Abstract

Most of fish species exhibit striking sexual dimorphism, particularly during growth. There are also sexual dimorphisms of internal organs and biological functions, including those of intestinal microbiota, which likely plays a key role in growth. In this study, the growth and intestinal microbiota of the female, male, and all-female *Nibea albiflora* (yellow drums) were comprehensively analyzed. The caged culture female and all-female yellow drums showed higher growth rates than males. A further analysis of the intestinal microbiota showed a significant difference in diversity between females and males in the summer, whereas there were no significant differences in the diversity and richness between females and males in the winter. In contrast, a significant difference in richness was observed between all-female and male fish, regardless of the season. Although the main composition of the intestinal microbiota showed no significant sex differences, the community structure of the intestinal microbiota of yellow drums did. Furthermore, the correlations between intestinal microbial communities are likely to be influenced by sex. The ecological processes of the intestinal microbial communities of the yellow drums showed clear sexual dimorphism. Further network analysis revealed that, although the main components of the network in the intestinal microbiota of female, male, and all-female fish were similar, the network structures showed significant sex differences. The negative interactions among microbial species were the dominant relationships in the intestinal ecosystem, and Bacteroidetes, Firmicutes, and Proteobacteria were identified as the functional keystone microbes. In addition, the functional pathways in the intestinal microbiota of yellow drums showed no significant sexual or seasonal differences. Based on the findings of this study, we gain a comprehensive understanding of the interactions between sex, growth, and intestinal microbiota in yellow drums.

## Introduction

Trillions of microbes that form a complex microbial community live in the intestines of organisms and are referred to as the intestinal microbiota. The intestinal microbiota is regarded as an external organ of the host (O’Hara and Shanahan, 2006; Sommer and Bäckhed, 2013), and has largely independent functional genetics for synthesizing enzymes, vitamins, and short-chain fatty acids (Ramakrishna, 2007; Larsen et al., 2014). Thus, the metabolic activities of the intestinal microbiota play key roles in maintaining the health and homeostasis of the host. The homeostasis of intestinal microbiota in turn, is not only related to the balance of diversity and richness of the microbial community, but also to interactions among species (Coyte et al., 2015; Yang et al., 2019a). Microbe-microbe interactions play vital roles in maintaining the stability and balance of the intestinal microbiota and in achieving systematic functions (Cordero and Datta, 2016; Widder et al., 2016). However, the composition of intestinal microbiota is highly malleable. The structure of the intestinal microbial community can be altered by factors such as diet (Smith et al., 2015; Duan et al., 2018; Yang et al., 2019b), habitat (Wu et al., 2018), host genetics (Rothschild et al., 2018), and so on.

Sexual dimorphism is among the most striking phenomena across various species (Fairbairn et al., 2007), particularly in fish. The sexually dimorphic growth of fish encouraged the production of mono-sex cultures in aquaculture. Sexual dimorphism can be observed in external and internal organs as well as biological functions, including the immune system and intestinal microbiota (Org et al., 2016; Strickland et al., 2021). Moreover, studies have shown that differences in immunity with respect to sex, could be a result of sex-specific variations in the intestinal microbiota (Flak et al., 2013; Klein and Flanagan, 2016; Elderman et al., 2018; Vemuri et al., 2019). However, sex differences in the intestinal microbiota are driven by sex hormones (Markle et al., 2013), which also affect bacterial gene expression, virulence, and growth, which have impacts on host physiology (Neuman et al., 2015; Rizzetto et al., 2018). Therefore, a bidirectional interaction could be confirmed between sex hormones and intestinal microbiota. Up to now, these interactions have mostly been reported in mammals. Although Bolnick et al. (2014) showed that gut microbiota composition depends on interactions between host diet and sex in two kinds of fish, the sexual dimorphism of the intestinal microbiota and its interaction with growth are poorly understood in fish. Consequently, in this study, we investigated whether sexual dimorphism of intestinal microbiota exists in fish and its possible role in sexual dimorphic growth.

The yellow drum (*Nibea albiflora*) belongs to the family Sciaenidae, which is distributed mainly in the coastal areas of China, Korea, and Japan (Xu et al., 2017; Qin et al., 2019). In the past decade, the production and quality of these fish have declined sharply due to environmental pollution and overfishing. Consequently, the mariculture of the yellow drum grew rapidly in the coastal regions of China. Currently, this fish is one of the major mariculture fishes in China because of its high economic value and increasing demand (Dai et al., 2012; Meng et al., 2021). By the age of 15 months, female yellow drums had grown 30% faster than males when subjected to cage culture (Xu et al., 2010; Chen et al., 2017). Thus, the yellow drum showed sexual dimorphism in growth, and mono-sex yellow drum cultures could improve the efficiency and profitability of the aquaculture of this species. Subsequently, we employed a new strategy for the mass production of all-female populations by crossing neo-males with normal females (Xu et al., 2018). In the present study, we analyzed the growth and intestinal microbiota of male, female, and all-female yellow drums, to explore the sexual dimorphism in intestinal microbiota and to clarify the interactions between sexually dimorphic intestinal microbiota and growth. The composition, structure, diversity, bacterial taxa, network structure, and ecological processes of the intestinal microbiota were investigated among female, male, and all-female yellow drums. The results of this study will provide new insights into sexual dimorphism in fish and contribute to better maintenance of guidance for mono-sex cultures.

## Materials and Methods

### Experimental Animal

The yellow drums used in the present study were obtained from a hatchery at the research station of the Marine Fishery Institute of Zhejiang Province, Xishan Island, Zhoushan, China. Normal fish were produced by natural spawning, while the all-female fish were obtained according to the method reported by Xu et al. (2018), by crossing neo-males (XX♂) and females. Normal and all-female fish were produced at the same time (20 May 2019), and the female brooder was from the same broodstock. Juvenile yellow drums (aged at 2 months) were delivered to the cages in the coastal region of Dengbu Island (29°52′21″N, 122°18′56″E). The water quality of the cultured region was shown in Supplementary Table 1. The same number of normal fish and all-female fish were placed in separate cages. After 1 month of acclimatization, the normal fish (female and male) were marked with red fluorescence on the back of the fish, and the all-female fish were marked with green fluorescence. The fish were weighed, and the initial weights of the normal fish and all-female fish were 26.56 ± 1.09 g and 26.98 ± 1.16 g, respectively. Then, the same number (500 individuals per cage) of the marked individuals for each fluorescence were pooled in the same cage (3 × 3 × 3 m) (Supplementary Figure 1). The fish were fed 2 – 3 times daily using commercial feed, and its ingredients were shown in Supplementary Table 2. The culture of the yellow drums in 15 cages was initiated in July 2019 and terminated in July 2021.

### Growth Indices

The initial weights of normal and all-female fish were noted at the beginning of the experiment. Body weight (BW), body length (BL), and total length (TL) were measured at 210, 434, 562, and 750 days post-hatch (dph), respectively, from 30 randomly selected individuals. The whole viscera weight was measured at 434 and 562 dph.

Fulton’s body condition factor K was calculated by using Fulton’s condition factor equation (Bagenal and Tesch, 1978):


$$K=\frac{10^{3}BW}{BL^{3}}$$

where BW represents body weight (g), and BL represents body length (cm).

The daily growth coefficient (DGC) was determined from the body weight data of each sample (Faggion et al., 2021). The formula was the following:

$$DGC=\frac{BW_{f}^{\frac{1}{3}}-BW_{i}^{\frac{1}{3}}}{t}\times 100$$

where BW_f_ represents the body weight at each time point, BW_i_ represents the initial weight, and t represents the number of days.

### Intestinal Sample Collection

Intestinal samples were collected according to the collection method reported by Wei et al. (2018), with some modifications. Eight cages were randomly chosen for sample collection. After a fasting period of 24 h, 15 normal fish were randomly collected from each cage and euthanized with an overdose of 3-aminobenzoic acid ethyl ester methanesulfonate (Sigma-Aldrich, St. Louis, MO, United States). Then, the skin surface of the fish was sterilized with 70% ethanol to reduce contamination. Subsequently, the sex of the fish was determined by dissection. Three female and three male individuals (similar in size) were chosen from the 15 individuals in each cage. The middle-intestine was then excised and transferred to a 10 mL aseptic tube. The middle-intestine contents of three individuals from the same cage were pooled as a single sample. After collection, the samples were immediately stored at −80°C. Samples of all-female fish were collected in the same way. At 434 dph on 17 July (summer), 24 samples were collected, and another batch of 24 samples was collected at 562 dph on 22 Nov. 2020 (winter). All samples were divided into the following six groups:

CS group: female fish samples from the summer;

CW group: female fish samples from the winter;

XS group: male fish samples from the summer;

XW group: male fish samples from the winter;

QS group: all-female fish samples from the summer;

QW group: all-female fish samples from the winter.

The experiment was implemented in strict accordance with the recommendations of the ethical principles of the Experimental Animal Welfare Ethics Committee of China. All procedures were performed in accordance with the Guide for the Care and Use of Laboratory Animals from Zhejiang Ocean University. All surgeries were performed under 3-aminobenzoic acid ethyl ester methanesulfonate anesthesia, and all efforts were made to minimize the suffering of yellow drums.

### DNA Extraction and 16S rRNA Gene Sequencing

The total DNA of each sample was extracted using the DNeasy PowerSoil Pro Kit (QIAGEN, Hilden, Germany). The total DNA concentration was quantified using a NanoDrop ND2000 spectrophotometer (Thermo Scientific, Waltham, MA, United States). All DNA samples were placed in a dry ice box and sent to Shanghai Oe Biotech Co., Ltd. (Shanghai, China) for Illumina sequencing on the MiSeq platform. The V3 + V4 regions of 16S rRNA were amplified using the barcoded fusion primers of 343F (5′-TACGGRAGGCAGCAG-3′) and 798R (5′-AGGGTATCTAATCCT-3′).

### Bioinformatics Analysis

Raw sequencing data were pretreated using QIIME software (v. 1.8.0; Caporaso et al., 2010). Paired-end reads were preprocessed using Trimmomatic software (v. 0.35) to obtain high-quality sequences. Paired-end reads were merged using FLASH software (v. 1.2.11) with overlapping length > 10 bp and a maximum mismatch rate < 0.2. UCHIME software (v. 2.4.2) was used to discard the chimeric sequences. The valid tags were clustered to generate operational taxonomic units (OTUs) using Vsearch software (v. 2.4.2) with a 97% similarity cutoff. The OTUs were annotated and classified using the Silva database (v. 123).

An UpSet plot was constructed to explore the distribution of OTUs and the composition of the shared OTUs in all groups using R (v. 4.1.0) with the UpSet package. Four α-diversity metrics were calculated namely: the Chao1 estimator, Observed_species for species richness, and phylogenetic diversity (PD) and Shannon index for diversity, using QIIME software. Non-metric multi-dimensional scaling analysis (NMDS) based on Jaccard distance was also performed using QIIME software. Permutational multivariate analysis of variances (PERMANOVA) was implemented to determine the dissimilarity of the microbial community composition among female, male, and all-female fish based on Bray-Curtis and Jaccard distance using the vegan package in R (v. 4.1.0). The Mantel test was used to assess the correlations with one another among the 10 most abundant classes using the ggcor package in R. The method of categorizing bacterial taxa was based on the report of Dai et al. (2016). Six categories based on their range of abundance: (i) rare taxa (RT), OTUs with abundance ≤ 0.1% in all samples; (ii) abundant taxa (AT), OTUs with abundance ≥ 1% in all samples; (iii) moderate taxa (MT), OTUs with abundance between 0.1 and 1% in all samples; (iv) conditionally rare taxa (CRT), taxa with abundance < 1% in all samples and ≤ 0.1% in some samples; (v) conditionally abundant taxa (CAT), taxa with abundance > than 0.1% in all samples and ≥ 1% in some samples but never rare (≤ 0.1%); (vi) conditionally rare or abundant taxa (CRAT), taxa with abundance varying from rare (≤ 0.1%) to abundant (≥ 1%).

### Null Model Analysis and Ecological Processes in the Assembly of the Intestinal Microbial Communities

Null model analysis was performed to explore the relative importance of stochastic and deterministic processes in the assembly of the intestinal microbial communities of the yellow drums. The null model analysis was performed using the vegan and parallel packages in R (v. 4.1.0), according to the method reported by Zhang Z. et al. (2019). The Bray-Curtis distance was used as the dissimilarity metric (D_obs_) across all communities, ranging from 0 to 1. The observed similarity (S_obs_) across the actual communities was complementary to the dissimilarity, that is, Sobs = 1 - Dobs. The null model algorithm was used to obtain the randomly expected similarity (E_exp_) of null expected communities (Chase et al., 2011; Stegen et al., 2013). Significant differences between the observed and expected similarities were analyzed using permutational multivariate analysis of variance (PERMANOVA) analysis. If ecological drift (e.g., stochastic colonization and extinction) and possible priority effects leading to multiple stable equilibria play predominant roles in determining community composition, the observed similarity will be statistically indistinguishable from the random null expectation (Zhou et al., 2014). By contrast, if community assembly is primarily shaped by deterministic processes, the observed similarity will be significantly higher than the random null expectation (Chase, 2007; Zhou et al., 2014).

The β nearest-taxon index (βNTI) and Raup-Crick (RC_bray_) were calculated according to the report by Zhang Z. et al. (2019) using R (v. 4.1.0) with the picante, ape, and parallel packages. βNTI is based on a null model test to evaluate the difference between the observed βMNTD (β mean nearest-taxon distance) and the mean of the null distribution (Stegen et al., 2013). βNTI values > + 2 or < −2 indicate that a pair of communities is regulated mainly by homogeneous selection (βNTI < −2) or heterogeneous selection (βNTI > + 2). In addition, RC_bray_ was used to divide the remaining pairwise comparisons with | βNTI| < 2. A value of RC_bray_ > + 0.95 is treated as the homogenizing dispersal, whereas a value of RC_bray_ < −0.95 represents the dispersal limitation (Stegen et al., 2013, 2015). The value of | RCbray| < 0.95 is considered as undominated, including weak selection, weak dispersal, diversification, and drift (Stegen et al., 2013; Zhou et al., 2014; Ning et al., 2020).

### Ecological Network Analysis of the Intestinal Microbiota

Phylogenetic molecular ecological networks (pMENs) were constructed using the random matrix theory-based interface approach in the Molecular Ecological Network Analysis pipeline (MENA^1^) (Deng et al., 2012). A network was naturally split into modules using the fast greedy modularity optimization method (Newman, 2004). Cytoscape software (v. 3.8.0) was used to delineate the ecological network. One node represented an OTU, corresponding to the microbial population. Nodes with different colors signified different phyla. Blue edges represent a positive interaction between two individual nodes, whereas red edges indicate a negative interaction.

The topological roles of nodes are divided into four types: peripherals, connectors, module hubs, and network hubs (Deng et al., 2012). Nodes with Zi < 2.5 and Pi < 0.6 were treated as peripherals. Nodes with Zi > 2.5 and Pi < 0.6 belonged to module hubs. Nodes with Zi < 2.5 and Pi > 0.6 were treated as connectors. Nodes with Zi > 2.5 and Pi > 0.6 served as network hubs.

### Functional Predictions of Intestinal Microbiota

Based on composition of the intestinal microbiota, Phylogenetic Investigation of Communities by Reconstruction of Unobserved States (PICRUSt) was used to predict the functional profiles (Langille et al., 2013). Functional pathways were annotated using the Kyoto Encyclopedia of Genes and Genomes (KEGG) database. NMDS based on Bray-Curtis distance was performed using R (v. 4.1.0) with the vegan package, which was applied to reflect changes in functional pathways among samples. Analysis of similarity (ANOSIM) was used to test the dissimilarity of the functional pathway composition among six groups based on the Jaccard distance method using the vegan package in R (v. 4.1.0). The raw sequencing data are available at the Sequence Read Archive (SRA) of the NCBI under the accession number PRJNA758491.

### Statistical Analysis

The differences in the α-diversity metrics were analyzed based on a two-way ANOVA test. One-way ANOVA and the *post hoc* Duncan test were performed to analyze the differences in the growth indices, DGC, and K. Before statistical analyses, data were checked for normality of distribution and homogeneity of variance using the Kolmogorov-Smirnov test and Levene’s test, respectively. When the raw data did not follow the normal distribution and/or homogeneous variances, Kruskal-Wallis rank-sum tests were performed. The differences between groups at the phylum, class, and genus levels were analyzed by using Kruskal-Wallis rank-sum tests. Significant differences were set at *P* < 0.05.

## Results

### Growth Indices, Fulton’s K Condition Factor and Daily Growth Coefficient

**FIGURE 1:**
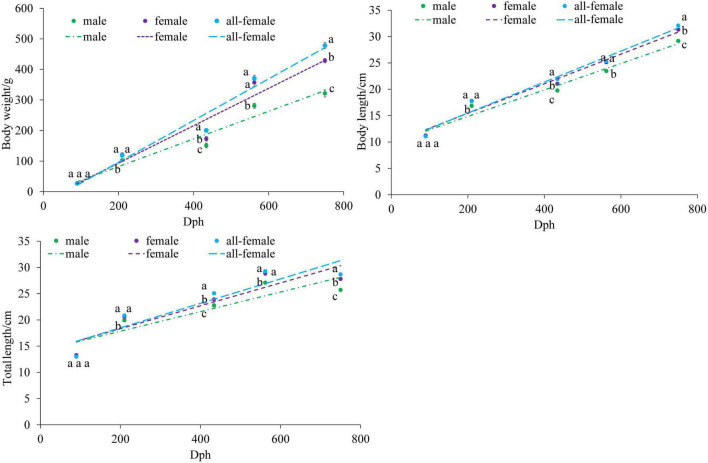
Growth curves of the body weight, body length, and total length of *Nibea albiflora* male, female, and all-female fish, during an investigation of the composition of the intestinal microbiota. Data with different letters at the same time point represented significant differences (*P* < 0.05).

The growth indices of the female, male, and all-female yellow drums were determined, and the results of Student’s *t*-test showed no significant difference in initial weight between normal fish (male and female) and all-female fish (*P* > 0.05) (Figure 1). However, at 210, 434, 562, and 750 dph, the body weights of the female and all-female fish were significantly higher than that of the male fish (*P* < 0.05). Furthermore, the body weight of the all-female fish was the highest at 434, 562, and 750 dph. At 434 and 750 dph, the body weights of the all-female fish were significantly higher than that of the female fish (*P* < 0.05). Body length and total length showed a similar pattern to that of the body weight.

Significant differences (*P* < 0.05) in the DGC were observed between males and females at 210, 434, 562, and 750 dph (Table 1). The DGC values of the all-female fish were significantly higher than that of males at 434, 562, and 750 dph (*P* < 0.05). Furthermore, the DGC value of the all-female fish was significantly higher than that of normal females at 750 dph (*P* < 0.05), and no significant differences (*P* > 0.05) in DGC were observed between females and all-female fish at 210, 434, and 562 dph. In addition, females and all-female fish were in better body condition than the males at 750 dph, with a significant difference in Fulton’s K condition factor. At 90, 210, 434, and 562 dph, the Fulton’s K condition factor was comparable among the male, female, and all-female fish.

**TABLE 1:** Daily growth coefficient and Fulton’s K condition factor.

Age (dph)	DGC ± SE			Fulton’s K ± SE		
	Male	Female	All-female	Male	Female	All-female
90 (initial value)	–	–	–	18.45 ± 0.41^a^ (normal fish)		19.74 ± 0.59^a^
210	0.78 ± 0.03^a^	0.91 ± 0.04^b^	0.89 ± 0.05^ab^	21.90 ± 0.97^a^	21.79 ± 0.82^a^	21.24 ± 0.68^a^
434	0.52 ± 0.03^a^	0.61 ± 0.03^b^	0.65 ± 0.02^b^	19.47 ± 0.39^a^	18.54 ± 0.40^a^	18.98 ± 0.39^a^
562	0.63 ± 0.01^a^	0.72 ± 0.02^b^	0.73 ± 0.02^b^	21.68 ± 0.28^a^	22.41 ± 0.40^a^	22.97 ± 0.75^a^
750	0.51 ± 0.02^a^	0.60 ± 0.01^b^	0.64 ± 0.01^c^	12.89 ± 0.25^a^	13.95 ± 0.08^b^	14.45 ± 0.18^b^

*Normal fish included female and male fish. Fulton’s K condition factor ± standard error for each age. Daily growth coefficient (DGC) ± standard error for each age. The DGC and Fulton’s K were determined from 30 randomly selected individuals. Values (mean ± S.E.M.) with different letters at the same row mean significant difference with each other (P < 0.05).*

### Richness, Diversity, and Structure of Microbial Community

**FIGURE 2:**
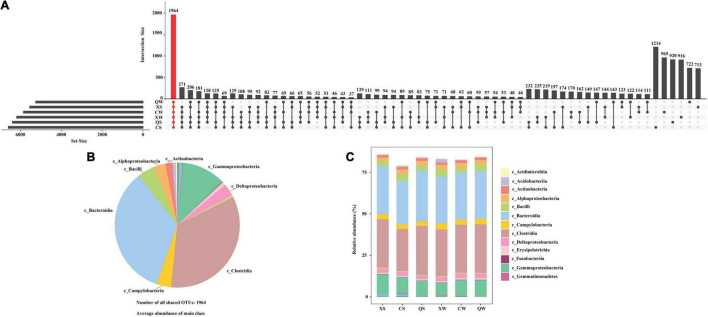
UpSet plot analysis of the intestinal microbiota *Nibea albiflora* male, female, and all-female fish at the operational taxonomic unit (OTU) level. X, C, and Q represented male, female, and all-female fish, respectively. Two seasons: summer (S) and winter (W). **(A)** UpSet plot analysis at the operational taxonomic unit (OTU) level; **(B)** dominant composition of shared OTUs at class level; **(C)** histogram showed the relative abundance of class in six groups.

A total of 3,348,327 (mean: 69,757 ± 1,623, min: 65,603, max: 72,778) effective tags were obtained from 48 samples using the Illumina MiSeq sequencing platform. The rarefaction curves of all the samples indicated that the number of tags was sufficient for analyzing the intestinal microbiota samples (Supplementary Figure 2). A total of 1,964 OTUs were shared by the six groups, and the highest number of unique OTUs was observed in the CS group (Figure 2A). In addition, the shared OTUs were mainly clustered into eight classes (relative abundance > 1.0%): Bacteroidia (28.44 ± 1.37%), Clostridia (28.30 ± 1.54%), Gammaproteobacteria (9.34 ± 1.13%), Bacilli (3.72 ± 0.69%), Campylobacteria (3.14 ± 0.30%), Deltaproteobacteria (2.66 ± 0.25%), Alphaproteobacteria (2.43 ± 0.34%) and Actinobacteria (1.46 ± 0.11%) (Figure 2B). Bacteroidia, Clostridia and Gammaproteobacteria were also the dominant classes in all groups (Figure 2C).

Significant differences in the Shannon and PD indices were observed among the male, female, and all-female fish (Shannon: *F*_2_,_48_ = 4.149, *p* = 0.022; PD: *F*_2_,_48_ = 4.272, *p* = 0.020). Meanwhile, a significant interaction in the Shannon and PD indices was observed between sex and season (Shannon: *F*_2_,_48_ = 4.296, *p* = 0.019; PD: *F*_2_,_48_ = 9.210, *p* < 0.001). No significant differences in the Chao1 and Observed_species indices were observed among males, females, and all-female fish (Chao1: *F*_2_,_48_ = 1.694, *p* = 0.195; Observed_species: *F*_2_,_48_ = 0.325, *p* = 0.571) (Table 2).

**TABLE 2:** Comparison of α-diversity metrics in groups and stages based on two-way ANOVA test.

	Chao1		Observed_species		Shannon		PD	
Two-way ANOVA test	*F*	*P*	*F*	*P*	*F*	*P*	*F*	*P*
Sex (X, C, Q)	1.694	0.195	0.325	0.571	4.149	0.022*	4.272	0.020*
Season (S, W)	0.132	0.718	2.693	0.078	2.309	0.135	10.756	0.002**
Sex * Season	0.999	0.376	1.404	0.255	4.296	0.019*	9.210	0.000***

The X, C and Q are respectively represented male, female, all-female fish. Two seasons: summer (S) and winter (W). PD, Phylogenetic diversity. ***Difference is significant at 0.001 level. **Difference is significant at 0.01 level. *Difference is significant at 0.05 level.

**FIGURE 3:**
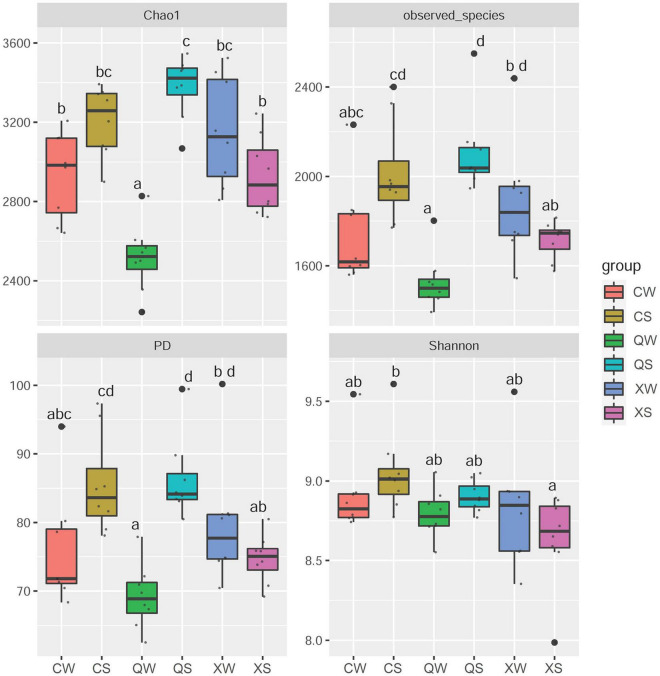
α-diversity metrics (Observed_species, Chao1, Shannon, and PD indices) in all groups of *Nibea albiflora* male, female, and all-female fish, during an investigation of the composition of the intestinal microbiota. PD: Phylogenetic diversity. X, C, and Q represented male, female, and all-female fish, respectively. Two seasons: summer (S) and winter (W). After running an ANOVA, *post hoc* Duncan tests were conducted to analyze the differences in the α-diversity metrics among the female, male, and all-female fish. Data with different letters at the column indicated significant differences.

Further *post hoc* Duncan tests revealed that the PD, Shannon, and Observed_species indices in the CS group were significantly higher than those in the XS group (*P* < 0.05) (Figure 3). However, there were no significant differences observed between the CW and XW groups (*P* > 0.05). In addition, there were no significant differences between the CS and QS groups in all α-diversity metrics (*P* > 0.05). Furthermore, there were no significant differences between the CS and QS groups seen in any of the α-diversity metrics (*P* > 0.05). The PD, Chao1, and Observed_species indices in the QS group were significantly higher than those in the XS groups (*P* < 0.05). However, the PD, Chao1, and Observed_species indices in the QW group were significantly lower than those in the XW group (*P* < 0.05). No significant difference was observed in the Shannon index among the XS, QS, XW, and QW groups (*P* > 0.05). In addition, no significant differences in all α-diversity metrics were observed between the CS and CW groups (*P* > 0.05), or between the XS and XW groups (*P* > 0.05). However, the PD, Chao1, and Observed_species indices in the QS group were significantly higher than those in the QW group (*P* < 0.05).

In terms of β-diversity, NMDS based on Jaccard distance was used to evaluate microbial community structural changes. There was a clear crossover among males, females and all-female fish (Supplementary Figure 3A). PERMANOVA analysis was used to explore the variation in the microbial community structure among all groups (Table 3). This analysis showed that the community structures of the intestinal microbiota exhibited no sex-significant difference (*P* > 0.05). However, significant differences between the two seasons were observed among the three sexes (*P* < 0.05). Furthermore, ANOSIM, PERMANOVA, and MRPP analysis showed significant differences between each pair of groups (*P* < 0.05, Supplementary Table 3). The microbial communities belonging to the same group were more closely clustered with one another (Supplementary Figure 3B).

**TABLE 3:** Dissimilarity tests of the microbial community composition using PERMANOVA based on Bray-Curtis and Jaccard distance. PERMANOVA, permutational multivariate analysis of variances.

	Bray-Curtis		Jaccard	
PERMANOVA test	*F*	*P*	*F*	*P*
Sex (X, C, Q)	1.056	0.204	1.040	0.199
Season (S, W)	1.418	0.012*	1.259	0.014*
Sex * Season	1.414	0.072	1.089	0.066

The X, C and Q are respectively represented male, female, all-female fish. Two seasons: summer (S) and winter (W). *Difference is significant at 0.05 level.

### Bacterial Community Composition and Classification

**FIGURE 4:**
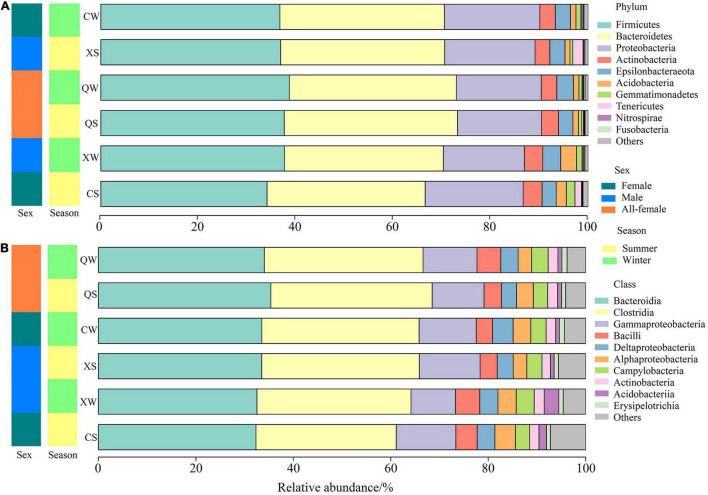
Relative abundances of different phyla **(A)** and classes **(B)** in the intestine of *Nibea albiflora* male, female, and all-female fish during an investigation of the composition of their intestinal microbiota.

Overall, sequences from all samples were identified as 36 prokaryotic phyla. The three most abundant phyla (relative abundance > 10% in each sample) in all samples were Firmicutes (36.95 ± 0.64%), Bacteroidetes (33.71 ± 0.56%), and Proteobacteria (18.24 ± 0.56%). These were the dominant phyla in all groups (Supplementary Table 4), and a similar composition of intestinal microbiota at the phylum level was observed among all groups (Figure 4A). In addition, the results of the Kruskal-Wallis rank-sum tests showed that there were no significant differences at the phylum level among all groups (*P* > 0.05). Additional information about the relative abundances at the phylum level among the six groups can be found in Supplementary Table 5.

At the class level, the relative abundances of the dominant classes were very similar among all groups (Figure 4B and Supplementary Table 6). In the 87 identified classes, 10 classes with *P* < 0.05 were identified in the six groups. The three most abundant OTUs in all groups were classified as Bacteroidia, Clostridia, and Gammaproteobacteria, and no significant differences in these classes were observed among the six groups (*P* > 0.05). Additionally, in the 1027 identified genera, 107 genera were identified with *P* < 0.05 in the six groups (Supplementary Table 7). The six major genera showed no significant differences among the six groups (Supplementary Figure 4 and Supplementary Table 7). Additional information about the relative abundances at the class- and genus levels can be found in the Supplementary Tables 6, 7.

Table 4 showed the bacterial taxa present in the overall OTUs in the six groups. Most OTUs in the six groups were distributed between RT and CRT, which accounted for more than 90% of the overall OTUs. Most OTUs in RT and CRT were affiliated to Alphaproteobacteria, Bacteroidia, Clostridia, Gammaproteobacteria and Deltaproteobacteria in the six groups (Supplementary Table 8). These results indicated that the global classification of bacterial OTUs exhibited no significant sex differences.

**TABLE 4:** Categories of bacterial taxa.

Categories	Male		Female		All-female	
	XS (5548)	XW (6182)	CS (6588)	CW (5846)	QS (6384)	QW (5250)
Rare taxa, RT/%	35.87 (1990)	33.19 (2052)	25.30 (1667)	29.06 (1699)	38.73 (2473)	26.76 (1405)
Abundant taxa, AT/%	0.13 (7)	0.02 (1)	0.03 (2)	0.14 (8)	0.02 (1)	0.13 (7)
Moderate taxa, MT/%	2.74 (152)	2.18 (135)	2.53 (167)	2.39 (140)	2.73 (174)	2.76 (145)
Conditionally rare taxa, CRT/%	61.01 (3385)	64.11 (3963)	71.80 (4730)	68.13 (3983)	58.22 (3717)	70.19 (3676)
Conditionally abundant taxa, CAT/%	0.16 (9)	0.36 (22)	0.18 (12)	0.26 (15)	0.27 (17)	0.21 (11)
Conditionally rare or abundant taxa, CRAT/%	0.09 (5)	0.15 (9)	1.52 (10)	0.02 (1)	0.03 (2)	0.11 (6)

*The X, C and Q are respectively represented male, female, all-female fish. Two seasons: summer (S) and winter (W). The number in bracket represented the number of OTUs.*

### Relationships Between α-Diversity Metrics and Growth Indices

The correlation between the α-diversity metrics and growth indices was analyzed using the Mantel test (Supplementary Figure 5), and the results revealed that there were no significant correlations between growth indices (body length, total length, body weight, and whole viscera weight) and α-diversity metrics (*P* > 0.05).

### Relationships Between Microbial Communities in the Intestinal Microbiota of the Female, Male, and All-Female Fish

**FIGURE 5:**
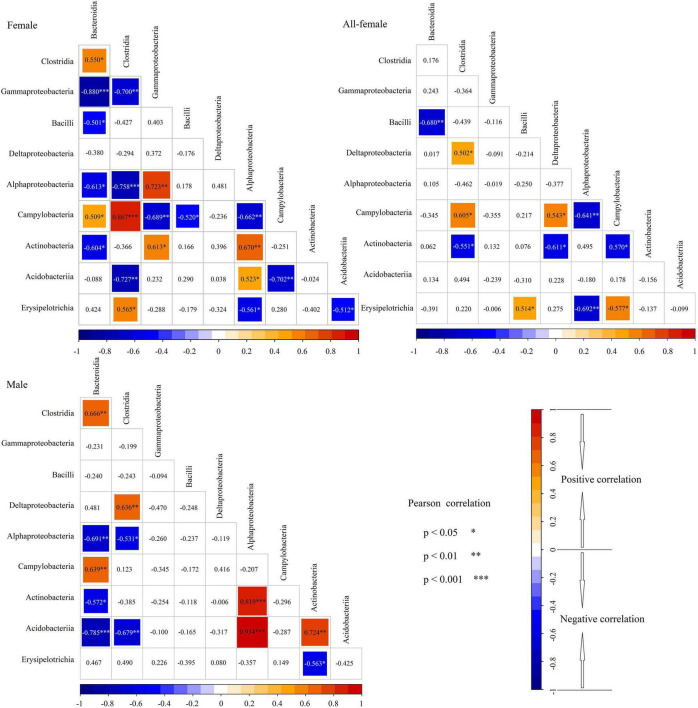
Relationships between microbial communities in the intestinal microbiota of *Nibea albiflora* female, male, and all-female fish during an investigation of the composition of their intestinal microbiota. Female: CS and CW groups; Male: XS and XW groups; All-female fish: QS and QW groups. Red in the cells indicated a positive correlation, while bule indicated a negative correlation. * Difference indicated by a significant correlation with *P* < 0.05. ** Difference indicated by a significant correlation with *P* < 0.01. *** Difference indicated by a significant correlation with *P* < 0.001.

Pearson correlation analysis showed that the correlations among the 10 most abundant classes were different for the female, male, and all-female fish (Figure 5). For instance, Bacteroidia and Clostridia were significantly and positively correlated in the intestinal microbiota of females and males (females: *P* < 0.05; males: *P* < 0.01). However, no significant correlation was observed between Bacteroidia and Clostridia in the intestinal microbiota of all-female fish (*P* > 0.05). In the intestinal microbiota of females and males, significant correlations were observed between Alphaproteobacteria and Bacteroidia (female: *P* < 0.05; male: *P* < 0.01), between Clostridia and Alphaproteobacteria (female: *P* < 0.001; male: *P* < 0.05), between Campylobacteria and Bacteroidia (female: *P* < 0.05; male: *P* < 0.01), between Actinobacteria and Bacteroidia (female: *P* < 0.05; male: *P* < 0.05), between Actinobacteria and Alphaproteobacteria (female: *P* < 0.01; male: *P* < 0.001), between Acidobacteriia and Clostridia (female: *P* < 0.01; male: *P* < 0.01), between Acidobacteriia and Alphaproteobacteria (female: *P* < 0.05; male: *P* < 0.001). However, the correlations between these classes were not found in the intestinal microbiota of all-female fish.

### Ecological Processes in the Assembly of the Intestinal Microbial Communities

The importance of deterministic and stochastic mechanisms in the assembly of intestinal microbial communities was analyzed using null model analysis (Table 5). The results of PERMANOVA revealed significant differences (CS group, *P* < 0.001; CW group, *P* < 0.001) between the observed similarity and null expected similarity, indicating that determinism of community assembly was more important than the stochasticity in the intestinal microbiota of the female fish. Meanwhile, in the XS, XW, QS, and QW groups, the observed similarities were significantly different from the null expected similarities (XS group, *P* < 0.01; XW group, *P* < 0.001; QS group, *P* < 0.001; QW group, *P* < 0.05), indicating that the dominant positions of stochasticity were observed in the assembly of the intestinal microbial communities of the male and all-female fish.

**TABLE 5:** Significance test of the similarity between the intestinal microbial communities of the yellow drums and null model simulations, and βNTI and RC_Bray_ values based on weighted Bray-Curtis distances.

Bray-Curtis		Mean of observed similarity	Mean of null expected similarity	*F*	*P* ^a^	βNTI^b^	RC_bray_^c^	Ecological processes shaping biodiversity
Female	CS	0.648	0.245	39.003	**0.001**	4.416	0.996	Determinism; Heterogeneous selection
	CW	0.696	0.256	18.023	**0.001**	4.348	0.781	Determinism; Heterogeneous selection
Male	XS	0.689	0.280	4.827	**0.002**	−1.292	0.793	Stochasticity; Undominated
	XW	0.668	0.236	14.534	**0.001**	−1.849	0.917	Stochasticity; Undominated
All-female	QS	0.681	0.228	10.652	**0.001**	−0.100	0.998	Stochasticity; Dispersal limitation
	QW	0.696	0.283	7.115	**0.031**	−1.324	0.223	Stochasticity; Undominated

*The X, C and Q are respectively represented male, female, all-female fish. Two seasons: summer (S) and winter (W).*

*^a^Permutational multivariate analysis of variance (PERMANOVA) was conducted. P-values < 0.05 were in bold.*

*^b^βNTI (βnearest-taxon index) is based on a null model test of the phylogenetic β-diversity index βMNTD (β mean nearest-taxon distance).*

*^c^RC_Bray_ (modified Raup-Crick index) is based on a null model test of the Bray-Curtis taxonomic β-diversity index.*

β nearest-taxon index was applied to reveal the ecological processes of the assembly of the intestinal microbial communities in the CS, CW, XS, XW, QS, and QW groups. In the CS and CW groups, the diversity of the intestinal microbiota was shaped by the heterogeneous selection of deterministic processes (βNTI value > 2). In contrast, the assemblies of the intestinal microbial communities in the XS, XW, and QW groups were shaped by undominated of stochasticity processes. However, the diversity of the intestinal microbiota of the QS group was shaped by the dispersal limitation of stochastic processes.

### Ecological Network Patterns in the Intestinal Microbiota

Phylogenetic molecular ecological networks were constructed to explore the microbial interactions within the intestinal microbial communities (Supplementary Table 9). The overall topology indices revealed that all network connectivity distribution curves fitted well with the power-law model (*R*^2^ values from 0.709 to 0.789). This indicated that most nodes in the network had few neighbors, while few nodes had many neighbors. The indices of pMENs (e.g., average path length and average clustering coefficient) were significantly different among each group, indicating that the structures of microbial communities in these groups were notably different. The modularity values in the six networks ranged from 0.888 to 0.927, which were significantly higher than the modularity values in their corresponding randomized networks, indicating that the six networks appeared to be modular. In addition, the indices in the empirical networks were higher than those in their corresponding random networks, indicating that the six networks obtained displayed typical small-world characteristics. In addition, the complexity of the network can be measured by the average connectivity. The QS group showed the most complex network, followed by the CS, XW, XS, CW, and QW groups.

**FIGURE 6:**
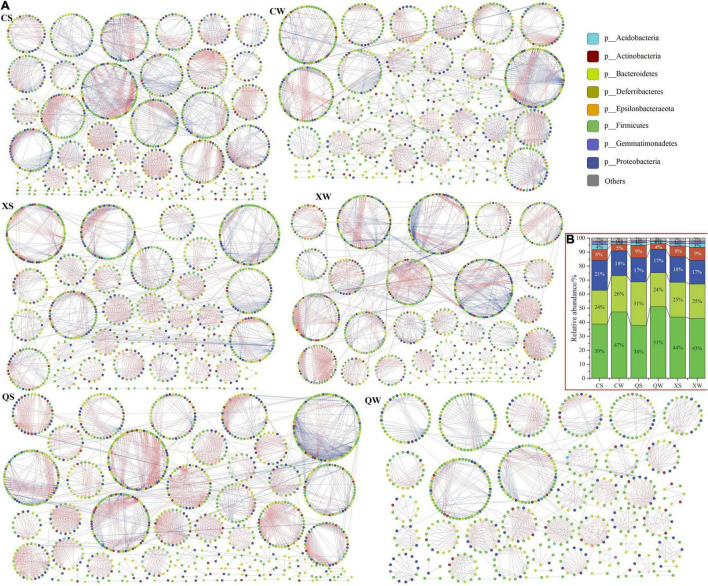
Ecological networks of the intestinal microbiota in the six groups of *Nibea albiflora* male, female, and all-female fish during an investigation of the composition of their intestinal microbiota **(A)**. Ecological network graph with submodule structure was obtained using the fast-greedy modularity optimization method. Each node indicated one OTU at the phylum level. Node colors indicated different major phyla. Blue edges indicated a positive interaction between two individual nodes, while red edges indicated a negative interaction. Main component in the six networks at the phylum level **(B)**. X, C, and Q represented male, female, and all-female fish, respectively. Two seasons: summer (S) and winter (W).

Subsequently, the overall pMENs were visualized using Cytoscape (v. 3.8.0; Figure 6A), and their compositions were shown in Figure 6B and Supplementary Table 10. The networks of CS, CW, QS, QW, XS, and XW consisted of 1072, 882, 1138, 679, 883, and 1007 nodes (OTUs), respectively. Most of the nodes were classified as Bacteroidetes (CS, CW, QS, QW, XS, and XW networks: 23.97, 25.85, 30.84, 24.15, 24.69, and 24.53%, respectively), Firmicutes (CS, CW, QS, QW, XS, and XW networks: 38.71, 47.28, 37.87, 51.10, 43.71, and 42.60%, respectively), and Proteobacteria (CS, CW, QS, QW, XS and XW networks: 21.36, 17.69, 17.14, 16.64, 18.23, and 16.98%, respectively), accounting for more than 80% in all groups. In the CS, CW, QS, QW, XS, and XW networks, the largest sub-modules had 81, 78, 97, 59, 86, and 107 nodes, respectively. Most nodes (> 40%) of the largest sub-modules in the six networks were classified as Firmicutes. Red edges represent negative interactions between nodes (OTUs), and blue edges represent positive interactions. Negative interactions (red edges) were dominant in the six networks (Figure 6A and Supplementary Table 10).

As shown in the *Z-P* plot, overall nodes from the six networks were divided into three types (peripherals, module hubs, and connectors), and most nodes (> 97%) were assigned to peripherals (Supplementary Figure 6). No nodes from the six networks were found in the network hubs. Most nodes that were divided into module hubs and connectors from the six networks were classified as Bacteroidetes, Firmicutes, and Proteobacteria (Supplementary Table 11).

### Functional Prediction of the Intestinal Microbiota

**FIGURE 7:**
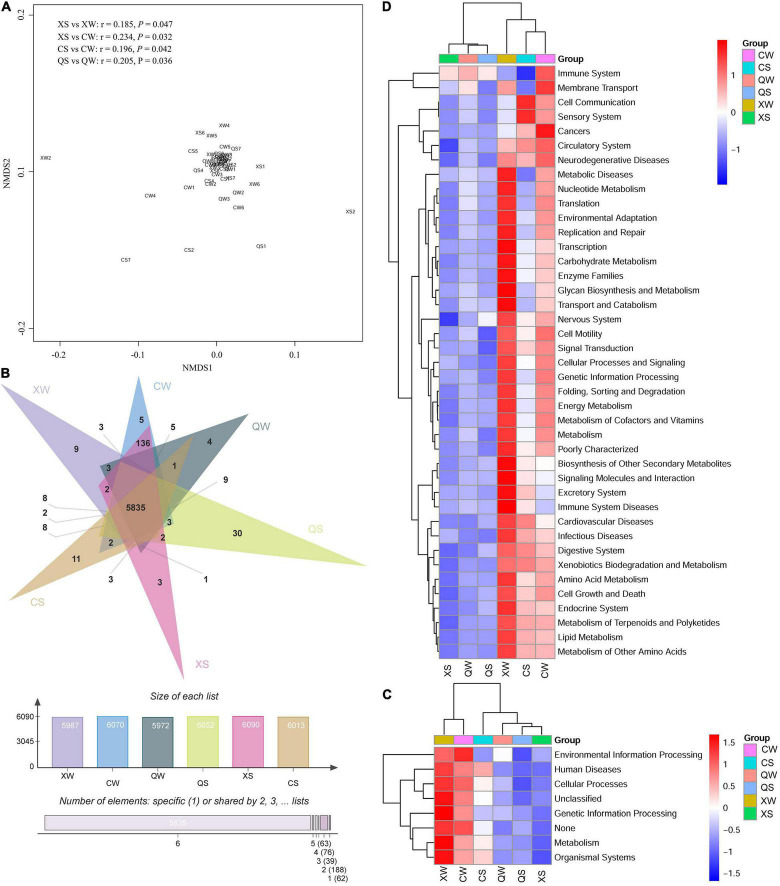
Non-metric multi-dimensional scaling analysis of the functional pathways in the six groups of *Nibea albiflora* male, female, and all-female fish during an investigation of the composition of their intestinal microbiota, based on Bray-Curtis distance **(A)**. Venn diagram of shared and unique functions among the six groups **(B)**. Heatmap showing the compositions of the functional pathways at the class-1 **(C)** and 2 **(D)** levels. X, C, and Q represented male, female, and all-female fish, respectively. Two seasons: summer (S) and winter (W).

Functional pathways of the intestinal microbiota in the CS, XS, QS, CW, XW, and QW groups were evaluated by predicting the metagenomes using PICRUSt. The NMDS plot showed that most samples from the six groups tended to cluster together (Figure 7A). Further ANOSIM analysis revealed that significant differences were observed between the XS and XW groups (*r* = 0.185, *p* = 0.047), XS and CW groups (*r* = 0.234, *p* = 0.032), CS and CW groups (*r* = 0.196, *p* = 0.042), and QS and QW groups (*r* = 0.205, *p* = 0.036). A total of 5835 functions were shared among the six groups (Figure 7B). As shown in Figure 7C, most of the functional genes were clustered to cellular processes, environmental information processing, genetic information processing and metabolism. The results of the Kruskal-Wallis rank-sum tests showed no significant differences in cellular processes, environmental information processing, genetic information processing and metabolism among the six groups (*P* > 0.05). Overall, functional genes from the six groups were clustered to 42 functional pathways at level 2 (Figure 7D), and no significant differences in those functional pathways were observed among the six groups (*P* > 0.05). Additional information about the functional pathways among the six groups can be found in Supplementary Table 12.

## Discussion

Sexual dimorphism is widespread in the aquatic animals. Previous studies have shown differences in the growth, metabolism, and phenotype of different sexes of aquatic animals (Li et al., 2012; Wang A. R. et al., 2018; Faggion et al., 2021). However, sexual dimorphism in the intestinal microbiota of aquatic animals is poorly understood. In this study, we analyzed the growth and intestinal microbiota of female, male, and all-female yellow drums. We found that the growth indices of females and all-female fish were significantly higher than those of males, and the growth indices of all-female fish were the highest for most of the sampling time. These data indicated that the growth rate of female and all-female fish was higher than that of males. Moreover, caged-culture all-female yellow drums have more benefits for improving the production of yellow drums. In addition, comparisons of DGC in the female, male, and all-female individuals showed the significant differences between the sexes. Faggion et al. (2021) also reported that a significant difference in the DGC was observed between female and male European sea bass at 96 and 103 dph, suggesting that physiological or biological changes occurred during this period. Previous studies have shown that growth was likely to be associated with the host’s intestinal microbiota (Wang et al., 2018). Exploring the differences in the intestinal microbiota between sexes would further improve our understanding of the underlying mechanism of sexual dimorphism in fish.

The structure of the intestinal microbiota is not only associated with the host species, but also with external environmental factors (Goodrich et al., 2014, 2016; Smith et al., 2015). Consequently, we pooled the male, female, and all-female individuals to eliminate the variations in the external environmental factors. We further performed a comprehensive comparison of the intestinal microbiota of yellow drums of different sexes. As revealed in this study, the Shannon and PD indices of the intestinal microbiota of the yellow drums showed significant sex differences. The results of *post hoc* Duncan tests showed no significant differences in α-diversity metrics in the intestinal microbiota of males or females between summer and winter. Similarly, α-diversity metrics in the intestinal microbiota of males showed no significant differences at the two seasons. However, the PD, Chao1, and Observed_species indices of the intestinal microbiota of all-female fish were significantly lower in the winter compared to the summer. These results indicated that the α-diversity in the intestinal microbiota of all-female fish was more susceptible to the change of seasons. In addition, the PERMANOVA revealed substantial variation in the microbial community structure of female, male, and all-female fish. Moreover, there were significant differences in the microbial community structure of female, male, and all-female fish between the two seasons. Further dissimilarity test results revealed that significant differences in the microbial community composition were observed among female, male, and all-female fish. These results corresponded to those of Strickland et al. (2021), who reported significant differences in the Shannon index and microbial composition of fecal microbiomes between male and female *Sigmodon fulviventer*. Ma and Li (2019) also observed a difference in the intestinal microbiota diversity between men and women.

Previous studies have shown that Bacteroidetes, Firmicutes, and Proteobacteria were the dominant phyla in the intestines of teleosts (Llewellyn et al., 2014; Colston and Jackson, 2016; Ramírez and Romero, 2017; Yang et al., 2019a). According to Wei et al. (2018) and Zhang C. et al. (2019), Firmicutes and Proteobacteria dominated the intestinal microbiota of yellow drums at the phylum level. In the present study, most OTUs in the intestinal microbiota of the female, male, and all-female fish were affiliated with Bacteroidetes, Firmicutes, and Proteobacteria, and no significant differences in the relative abundance of these three phyla were found among female, male and all-female fish. Similarly, Ma and Li (2019) reported that Bacteroidetes, Firmicutes, and Proteobacteria were the major phyla in the intestinal microbiota of men and women, and that no significant sex differences were observed. Furthermore, a similar composition of the intestinal microbiota at the phylum level was observed among female, male, and all-female fish. These results indicated that the composition of the intestinal microbiota of yellow drums exhibited no significant sex or seasonal differences.

*Bacteroides* play a key role in maintaining host homeostasis (Hooper and Gordon, 2001; Sears, 2005; Wu et al., 2007). For example, Bäckhed et al. (2005) reported that *B. thetaiotaomicron* imparts stability to the intestinal ecosystem through its capacity to turn to host polysaccharides when dietary polysaccharides become scarce. *Lactobacillus* is a potentially beneficial bacterium that could contribute to improving the growth and immunity of aquatic animals (Zheng et al., 2018). Strickland et al. (2021) reported that *Lactobacillus* was associated with protection against foreign infections. In the present study, *Bacteroides* and *Lactobacillus* were the dominant genera in the intestinal microbiota of female, male, and all-female fish and showed no significant sex differences.

Rare taxa served as a reservoir of species, greatly contributing to the quantification of species richness within a given community and functional genes (Jia et al., 2018). Changes in the abundance of rare species could affect ecosystem function (Jia et al., 2018). In the present study, RT and CRT were dominant in the female, male, and all-female fish, and the major categories of bacterial taxa exhibited no distinct sex differences.

The Mantel test showed no significant differences between α-diversity metrics and growth indices. As such, the correlations between α-diversity metrics and growth indices were not significantly different between the sexes. The results of Pearson correlation analysis revealed that the correlations among the 10 most abundant classes were distinctly different among female, male, and all-female fish. This indicated that the correlation between intestinal microbial communities could be influenced by sex.

The determinism and stochasticity mechanisms of the community assembly play key roles in shaping the composition and diversity of the microbial community (Zhang Z. et al., 2019). However, stochastic and deterministic processes in microbial succession are not alterable. For example, Dini-Andreote et al. (2015) reported that community composition was initially governed by stochasticity, but as succession proceeded, there was a progressive increase in deterministic selection correlated with increasing sodium concentration. Burns et al. (2016) reported that ecological processes in the assembly of the intestinal microbial communities varied during zebrafish development; in the larval and juvenile stages, stochastic processes played a dominant role in shaping the assembly of the intestinal microbial communities. However, the determinism of community assembly was more important than stochasticity in the intestinal microbiota of the zebrafish at the adult developmental stage. Wang L. et al. (2020), Wang Y. et al. (2020) reported that the assembly of the bacterial community of *Litopenaeus vannamei* larvae was overall governed by neutral processes (dispersal among individuals and ecological drift) at all stages. In the present study, the ecological processes of the intestinal microbial communities of yellow drums showed significant sex differences, but no significant differences were observed between seasons.

Ecological network analysis could provide another viewpoint for understanding complex intestinal microbiota. The stability of the ecological network in intestinal microbiota is not only connected to the diversity and composition of the intestinal microbiota, but also to the interactions between different species within the intestinal microbiota (Hooper and Gordon, 2001; Ley et al., 2006; Mazmanian and Lee, 2014). In terms of the properties of the ecological network, modularity is a very important index that delineates its resilience and stability (Olesen et al., 2007). In this study, higher modularity values were found in the six networks, indicating that the resilience and stability of the ecological network of the intestinal microbiota in female, male, and all-female fish were higher. Although the microbial compositions of the six networks tended to be similar, the structures of the six networks still showed differences. The predominant component in all networks was also the major component of the intestinal microbiota, indicating that the predominant microbial communities occupied an important position in the network. In a network, positive interactions might signify cooperation or complementation among species, while negative interactions might indicate competition, predation, or amensalism (Faust and Raes, 2012; Feng et al., 2017). Competitive relationships have been found to be prevalent in natural microbial communities (Foster and Bell, 2012) and are more conducive to maintaining the stability of networks (Coyte et al., 2015). Our data revealed that the microbial relationships were dominated by negative interactions in all six networks. In addition, clear differences in the number of OTUs, edges, and sub-modules were observed between the QS (1138 OTUs, 3060 edges, and 71 sub-modules) and QW networks (679 OTUs, 1350 edges, and 45 sub-modules). This indicated that the composition and structure of the ecological network of all-female fish were easily altered by the different seasons.

Species serving as module hubs or connectors can be regarded as functional keystone species in ecological networks (Olesen et al., 2007). In this study, most OTUs that served as module hubs or connectors of the six networks were affiliated with Bacteroidetes, Firmicutes, and Proteobacteria. Thus, Bacteroidetes, Firmicutes and Proteobacteria could be considered as functional keystone species in the ecological network of the intestinal microbiota of yellow drums, and their functional roles showed no sex and seasonal differences.

The intestine contains trillions of microbes that can shape the host’s metabolism and immune system (Rastelli et al., 2019). Previous studies have suggested that the metabolic function of the intestinal microbiota is associated with the host’s species and environmental factors (Visconti et al., 2019; Fan et al., 2020). However, the functional differences in the intestinal microbiota of aquatic animals between sexes remain unclear. In this study, more than 99.5% of the functional pathways of the intestinal microbiota were found in female, male, and all-female fish, and no significant differences in functional pathways at class-2 and 3 levels were observed among female, male, and all-female fish. These results indicated that the composition and relative abundance of functional pathways showed no significant differences between sexes and seasons.

## Conclusion

In the present study, sexual dimorphism in the growth of yellow drums was observed. Further intestinal microbiota analysis showed that there were significant differences in the richness of intestinal microbiota between males and all-females, regardless of the season. Correlations between microbial communities were likely influenced by sex. Further intestinal microbial network analysis revealed significant differences in network structures between sexes, indicating that the functions of the intestinal microbial communities might differ between the sexes. The findings of this study will contribute to the understanding of sexual dimorphism in aquatic animals and provide new insights into mono-sex aquaculture.

## Data Availability Statement

The datasets presented in this study can be found in online repositories. The names of the repository/repositories and accession number(s) can be found below: https://www.ncbi.nlm.nih.gov/, PRJNA758491.

## Ethics Statement

The animal study was reviewed and approved by Guide for the Care and Use of Laboratory Animals from Zhejiang Ocean University.

## Author Contributions

HL, LL, RC, SL, and DX contributed to material preparation, data collection, and analysis were performed. HL wrote the first draft of the manuscript. All authors commented on previous versions of the manuscript, read and approved the final manuscript, and contributed to the study conception and design.

## Conflict of Interest

The authors declare that the research was conducted in the absence of any commercial or financial relationships that could be construed as a potential conflict of interest.

## Publisher’s Note

All claims expressed in this article are solely those of the authors and do not necessarily represent those of their affiliated organizations, or those of the publisher, the editors and the reviewers. Any product that may be evaluated in this article, or claim that may be made by its manufacturer, is not guaranteed or endorsed by the publisher.

## Footnotes

1
http://ieg2.ou.edu/MENA/
