# Supplementary material for: Exploring Sexual Dimorphism in the Intestinal Microbiota of the Yellow Drum (Nibea albiflora, Sciaenidae)
Source: Front Microbiol. 2022 Jan 5;12:808285. doi: 10.3389/fmicb.2021.808285 (PMC8767002; doi:10.3389/fmicb.2021.808285)
Supplement: Supplementary file 1 [file Table_1.DOCX]

## Table 1 The water quality of the cultured region.

|  | OD/mg/L | pH | Sal | T |
| --- | --- | --- | --- | --- |
| Spring | 7.8 ± 0.4 | 7.2 ± 0.2 | 29.2 ± 0.9 | 19.9 ± 1.9 |
| Summer | 7.3 ± 1.2 | 7.1 ± 0.1 | 28.4 ± 0.5 | 28.0 ± 1.1 |
| Autumn | 6.5 ± 1.5 | 7.1 ± 0.1 | 28.3 ± 1.1 | 23.0 ± 3.2 |
| Winter | 6.8 ± 0.8 | 7.3 ± 0.1 | 28.0 ± 0.5 | 12.7 ± 3.2 |

Note: Data were showed as mean ± standard error (SEM). OD: average dissolved oxygen; Sal: average salinity; T: average temperature.
