# Supplementary material for: Exploring Sexual Dimorphism in the Intestinal Microbiota of the Yellow Drum (Nibea albiflora, Sciaenidae)
Source: Front Microbiol. 2022 Jan 5;12:808285. doi: 10.3389/fmicb.2021.808285 (PMC8767002; doi:10.3389/fmicb.2021.808285)
Supplement: Supplementary file 2 [file Table_2.DOCX]

## Table 2 Nutritional compositions of the commercial feed

| Composition/ (dry weight) | Percentage (%) |
| --- | --- |
| Crude protein | ≥ 49.0 |
| Crude fat | ≥ 8.0 |
| Crude fiber | ≤ 6.0 |
| Ash | ≤ 16.0 |
| Total phosphorus | ≥ 1.2 |
| Moisture | ≤11.0 |
| Lysine | ≥ 3.2 |

Note: The commercial feed was purchased from Zhejiang Han Bank Biotech Co., Ltd. (Huzhou, China)
