# Supplementary material for: Exploring Sexual Dimorphism in the Intestinal Microbiota of the Yellow Drum (Nibea albiflora, Sciaenidae)
Source: Front Microbiol. 2022 Jan 5;12:808285. doi: 10.3389/fmicb.2021.808285 (PMC8767002; doi:10.3389/fmicb.2021.808285)
Supplement: Supplementary file 3 [file Table_3.DOCX]

## Table 3 Dissimilarity tests of the microbial community composition using ANOSIM, PERMANOVA and MRPP based on the Jaccard distance. MRPP, Multiple Response Permutation Procedure; ANOSIM, analysis of similarities.

| Group | ANOSIM | PERMANOVA | MRPP |
| --- | --- | --- | --- |
|  | R *P* | F *P* | A *P* |
| XS vs CS | 0.561 0.002 | 1.393 0.001 | 0.691 0.001 |
| XS vs QS | 0.746 0.001 | 1.545 0.002 | 0.674 0.002 |
| CS vs QS | 0.655 0.001 | 1.617 0.001 | 0.680 0.001 |
| XW vs CW | 0.492 0.001 | 1.411 0.001 | 0.700 0.001 |
| XW vs QW | 0.658 0.002 | 1.554 0.001 | 0.702 0.001 |
| CW vs QW | 0.589 0.001 | 1.396 0.001 | 0.706 0.001 |
| XS vs XW | 0.537 0.001 | 1.379 0.001 | 0.689 0.002 |
| CS vs CW | 0.785 0.003 | 1.664 0.001 | 0.698 0.001 |
| QS vs QW | 0.819 0.002 | 1.906 0.001 | 0.687 0.001 |

The X, C and Q are respectively represented male, female, all-female fish. Two seasons: summer (S) and winter (W).
