# Supplementary material for: Exploring Sexual Dimorphism in the Intestinal Microbiota of the Yellow Drum (Nibea albiflora, Sciaenidae)
Source: Front Microbiol. 2022 Jan 5;12:808285. doi: 10.3389/fmicb.2021.808285 (PMC8767002; doi:10.3389/fmicb.2021.808285)
Supplement: Supplementary file 4 [file Table_4.DOCX]

## Table 4 Dominant phyla in all groups.

|  | XS | CS | QS | XW | CW | QW |
| --- | --- | --- | --- | --- | --- | --- |
| Firmicutes | 36.87 ± 0.80% | 34.06 ± 1.94% | 37.59 ± 0.80% | 37.66 ± 2.48% | 36.75 ± 1.26% | 38.73 ± 1.38% |
| Bacteroidetes | 36.66 ± 0.66% | 32.45 ± 2.05% | 35.58 ± 0.70% | 32.59 ± 2.18% | 33.76 ± 1.09% | 34.24 ± 2.16% |
| Proteobacteria | 18.55 ± 1.84% | 20.14 ± 2.02% | 17.21 ± 0.33% | 16.62 ± 0.81% | 19.57 ± 1.46% | 17.38 ± 0.91% |

The X, C and Q are respectively represented male, female, all-female fish. Two seasons: summer (S) and winter (W). Data expressed as a mean ± S. E. M.
