# Supplementary material for: Exploring Sexual Dimorphism in the Intestinal Microbiota of the Yellow Drum (Nibea albiflora, Sciaenidae)
Source: Front Microbiol. 2022 Jan 5;12:808285. doi: 10.3389/fmicb.2021.808285 (PMC8767002; doi:10.3389/fmicb.2021.808285)
Supplement: Supplementary file 8 [file Table_8.DOCX]

## Table 8 The main composition of rare taxa and conditionally rare taxa in gut microbiota of the male, female and all-female fish.

| Categories | Phylum level | Male | | Female | | All-female | |
| --- | --- | --- | --- | --- | --- | --- | --- |
|  |  | XS | XW | CS | CW | QS | QW |
| rare taxa, RT/% | p__Bacteroidetes | 32.71 (651) | 37.87 (777) | 38.39 (640) | 37.67 (640) | 39.39 (974) | 37.30 (524) |
|  | p__Firmicutes | 27.54 (548) | 28.27 (580) | 28.31 (472) | 32.43 (551) | 25.11 (621) | 31.53 (443) |
|  | p__Proteobacteria | 9.00 (179) | 6.34 (130) | 6.60 (110) | 6.06 (103) | 6.96 (172) | 7.47 (105) |
|  | p__Proteobacteria | 6.19 (123) | 5.85 (120) | 5.34 (89) | 4.71 (80) | 5.38 (133) | 5.69 (80) |
|  | p__Proteobacteria | 4.22 (84) | 3.46 (71) | 4.08 (68) | 3.41 (58) | 4.41 (109) | 2.92 (41) |
| conditionally rare taxa, CRT/% | p__Bacteroidetes | 33.00 (1117) | 28.77 (1140) | 29.60 (1400) | 29.53 (1176) | 29.46 (1095) | 32.67 (1201) |
|  | p__Firmicutes | 30.93 (1047) | 27.73 (1099) | 24.95 (1180) | 25.81 (1028) | 30.78 (1144) | 29.87 (1097) |
|  | p__Proteobacteria | 9.69 (328) | 10.50 (416) | 10.51 (497) | 11.93 (475) | 10.87 (404) | 10.09 (371) |
|  | p__Proteobacteria | 4.79 (162) | 5.70 (226) | 5.64 (267) | 5.95 (237) | 5.17 (192) | 4.35 (160) |
|  | p__Proteobacteria | 3.75 (127) | 3.99 (158) | 5.62 (266) | 4.97 (198) | 3.69 (137) | 3.48 (128) |

The X, C and Q are respectively represented male, female, all-female fish. Two seasons: summer (S) and winter (W). The number in bracket represented the number of OTUs.
