# Supplementary material for: Exploring Sexual Dimorphism in the Intestinal Microbiota of the Yellow Drum (Nibea albiflora, Sciaenidae)
Source: Front Microbiol. 2022 Jan 5;12:808285. doi: 10.3389/fmicb.2021.808285 (PMC8767002; doi:10.3389/fmicb.2021.808285)
Supplement: Supplementary file 9 [file Table_9.DOCX]

## Table 9 Topological properties of the empirical phylogenetic molecular ecological networks of gut microbiota and their associated random networks.

|  | Empirical networks | | | | | | | Random networks ^a^ | | |
| --- | --- | --- | --- | --- | --- | --- | --- | --- | --- | --- |
| groups | Similarity threshold (s_t_) | Network size (n) | R^2^ of power law | Average connectivity (*avg*K) | Average path length (GD) ^b^ | Average clustering coefficient (*avg*CC) | Modularity (Module No.) | Average path length (GD) | Avg. clustering coefficient (*avg*CC) | Modularity (Module No.) |
| CS | 0.95 | 1072 | 0.759 | 5.121 | 8.691^c^ | 0.119^d^ | 0.913(66)^e^ | 4.274±0.016 | 0.007±0.001 | 0.432±0.004 |
| XS | 0.95 | 883 | 0.709 | 4.766 | 8.570^c^ | 0.147^d^ | 0.898(42)^ef^ | 4.354±0.019 | 0.007±0.002 | 0.456±0.004 |
| QS | 0.95 | 1138 | 0.778 | 5.378 | 8.267^c^ | 0.103^d^ | 0.897(72)^eg^ | 4.170±0.016 | 0.007±0.001 | 0.417±0.003 |
| CW | 0.95 | 882 | 0.771 | 4.676 | 8.838^c^ | 0.130^d^ | 0.893(53)^eg^ | 4.341±0.019 | 0.008±0.002 | 0.462±0.004 |
| XW | 0.95 | 1007 | 0.769 | 5.098 | 7.967^c^ | 0.158^d^ | 0.888(46)^efg^ | 4.234±0.016 | 0.007±0.002 | 0.434±0.004 |
| QW | 0.95 | 679 | 0.789 | 3.976 | 10.352^c^ | 0.139^d^ | 0.927(46)^efg^ | 4.636±0.028 | 0.007±0.002 | 0.519±0.004 |
| The X, C and Q are respectively represented male, female, all-female fish. Two seasons: summer (S) and winter (W).  a. Random networks were generated by rewiring all nodes and links corresponding to empirical networks 100 times.  b. GD, geodesic distance.  c. Significant difference (*P*<0.01) in average path between any two groups based on Student’s *t* test.  d. Significant difference (*P*<0.01) in Average clustering coefficient between any two groups based on Student’s *t* test.  e. Significant difference (*P*<0.01) in modularity for CS group with other five groups based on Student’s *t* test. | | | | | | | | | | |

f. Significant difference (*P*<0.01) in modularity for XS group with other two groups based on Student’s *t* test.

g. Significant difference (*P*<0.01) in modularity for QW group with other three groups based on Student’s *t* test.
