# Supplementary material for: Exploring Sexual Dimorphism in the Intestinal Microbiota of the Yellow Drum (Nibea albiflora, Sciaenidae)
Source: Front Microbiol. 2022 Jan 5;12:808285. doi: 10.3389/fmicb.2021.808285 (PMC8767002; doi:10.3389/fmicb.2021.808285)
Supplement: Supplementary file 10 [file Table_10.DOCX]

## Table 10 Different indexes of ecological network in six groups.

| Index | CS | CW | QS | QW | XS | XW |
| --- | --- | --- | --- | --- | --- | --- |
| p__Acidobacteria | 39 | 10 | 22 | 10 | 16 | 26 |
| p__Actinobacteria | 83 | 43 | 102 | 28 | 68 | 94 |
| p__Bacteroidetes | 257 | 228 | 351 | 164 | 218 | 247 |
| p__Chloroflexi | 0 | 0 | 0 | 0 | 0 | 1 |
| p__Cyanobacteria | 0 | 1 | 0 | 0 | 0 | 1 |
| p__Deferribacteres | 1 | 4 | 2 | 1 | 3 | 2 |
| p__Epsilonbacteraeota | 4 | 4 | 8 | 3 | 7 | 5 |
| p__Fibrobacteres | 1 | 1 | 0 | 0 | 1 | 1 |
| p__Firmicutes | 415 | 417 | 431 | 347 | 386 | 429 |
| p__Fusobacteria | 4 | 4 | 2 | 4 | 2 | 3 |
| p__Gemmatimonadetes | 26 | 8 | 16 | 3 | 13 | 19 |
| p__Latescibacteria | 1 | 0 | 0 | 0 | 0 | 0 |
| p__Nitrospirae | 2 | 2 | 3 | 2 | 3 | 1 |
| p__Patescibacteria | 2 | 0 | 0 | 1 | 0 | 0 |
| p__Proteobacteria | 229 | 156 | 195 | 113 | 161 | 171 |
| p__Spirochaetes | 2 | 1 | 0 | 1 | 0 | 0 |
| p__Tenericutes | 4 | 2 | 5 | 2 | 4 | 5 |
| p__Verrucomicrobia | 1 | 0 | 1 | 0 | 0 | 1 |
| p__Zixibacteria | 1 | 1 | 0 | 0 | 1 | 1 |
| Total number of OTUs | 1072 | 882 | 1138 | 679 | 883 | 1007 |
| Total number of Modules | 65 | 52 | 71 | 45 | 41 | 45 |
| The number of blue edges | 1248 | 949 | 1112 | 681 | 878 | 1124 |
| The number of red edges | 1497 | 1113 | 1948 | 669 | 1226 | 1443 |
| Total number of edges | 2745 | 2062 | 3060 | 1350 | 2104 | 2567 |
| The number of module hubs | 14 | 16 | 17 | 9 | 12 | 19 |
| The number of connectors | 8 | 1 | 3 | 1 | 5 | 0 |
