# Supplementary material for: Exploring Sexual Dimorphism in the Intestinal Microbiota of the Yellow Drum (Nibea albiflora, Sciaenidae)
Source: Front Microbiol. 2022 Jan 5;12:808285. doi: 10.3389/fmicb.2021.808285 (PMC8767002; doi:10.3389/fmicb.2021.808285)
Supplement: Supplementary file 11 [file Table_11.DOCX]

## Table 11 Topological roles of intestinal microbiota in the six groups.

| Treatment | Types | Phylogenetic associations |
| --- | --- | --- |
| CS | **Module hubs**  OTU48  OTU104  OTU232  OTU574  OTU687  OTU755  OTU937  OTU1062  OTU1522  OTU2091  OTU2297  OTU4479  OTU5081  OTU5931 | p__Proteobacteria  p__Firmicutes  p__Firmicutes  p__Bacteroidetes  p__Proteobacteria  p__Firmicutes  p__Firmicutes  p__Bacteroidetes  p__Firmicutes  p__Firmicutes  p__Proteobacteria  p__Proteobacteria  p__Acidobacteria  p__Firmicutes |
|  | **Connectors**  OTU1180  OTU244  OTU260  OTU263  OTU370  OTU3883  OTU8067  OTU900 | p__Actinobacteria  p__Firmicutes  p__Bacteroidetes  p__Firmicutes  p__Proteobacteria  p__Actinobacteria  p__Firmicutes  p__Bacteroidetes |
| CW | **Module hubs**  OTU170  OTU233  OTU260  OTU3183  OTU336  OTU384  OTU427  OTU45  OTU458  OTU641  OTU711  OTU762  OTU836  OTU95  OTU970  OTU979 | p__Firmicutes  p__Firmicutes  p__Bacteroidetes  p__Bacteroidetes  p__Actinobacteria  p__Bacteroidetes  p__Firmicutes  p__Proteobacteria  p__Proteobacteria  p__Firmicutes  p__Actinobacteria  p__Firmicutes  p__Proteobacteria  p__Proteobacteria  p__Bacteroidetes  p__Firmicutes |
|  | **Connectors**  OTU8867 | p__Firmicutes |
| QS | **Module hubs**  OTU1110  OTU1493  OTU1514  OTU1620  OTU19  OTU21  OTU2165  OTU249  OTU36  OTU3699  OTU3828  OTU4118  OTU428  OTU4548  OTU5711  OTU7  OTU7751 | p__Firmicutes  p__Firmicutes  p__Firmicutes  p__Proteobacteria  p__Firmicutes  p__Bacteroidetes  p__Bacteroidetes  p__Proteobacteria  p__Firmicutes  p__Firmicutes  p__Bacteroidetes  p__Actinobacteria  p__Firmicutes  p__Proteobacteria  p__Firmicutes  p__Proteobacteria  p__Bacteroidetes |
|  | **Connectors**  OTU2831  OTU7826  OTU8278 | p__Firmicutes  p__Actinobacteria  p__Firmicutes |
| QW | **Module hubs**  OTU1082  OTU213  OTU539  OTU687  OTU694  OTU695  OTU815  OTU869  OTU908 | p__Bacteroidetes  p__Bacteroidetes  p__Bacteroidetes  p__Proteobacteria  p__Firmicutes  p__Firmicutes  p__Firmicutes  p__Firmicutes  p__Firmicutes |
|  | **Connectors**  OTU1103 | p__Firmicutes |
| XS | **Module hubs**  OTU1108  OTU1510  OTU1522  OTU1708  OTU1929  OTU3586  OTU4118  OTU424  OTU52  OTU543  OTU762  OTU91 | p__Firmicutes  p__Bacteroidetes  p__Firmicutes  p__Proteobacteria  p__Firmicutes  p__Bacteroidetes  p__Actinobacteria  p__Firmicutes  p__Firmicutes  p__Bacteroidetes  p__Firmicutes  p__Proteobacteria |
|  | **Connectors**  OTU115  OTU3201  OTU715  OTU785  OTU851 | p__Firmicutes  p__Bacteroidetes  p__Proteobacteria  p__Firmicutes  p__Firmicutes |
| XW | **Module hubs**  OTU107  OTU11493  OTU125  OTU28  OTU403  OTU414  OTU433  OTU444  OTU458  OTU4701  OTU542  OTU543  OTU545  OTU572  OTU635  OTU721  OTU810  OTU82  OTU9654 | p__Firmicutes  p__Bacteroidetes  p__Bacteroidetes  p__Firmicutes  p__Firmicutes  p__Actinobacteria  p__Proteobacteria  p__Bacteroidetes  p__Proteobacteria  p__Bacteroidetes  p__Firmicutes  p__Bacteroidetes  p__Firmicutes  p__Bacteroidetes  p__Bacteroidetes  p__Firmicutes  p__Firmicutes  p__Firmicutes  p__Bacteroidetes |
