# Supplementary material for: Exploring Sexual Dimorphism in the Intestinal Microbiota of the Yellow Drum (Nibea albiflora, Sciaenidae)
Source: Front Microbiol. 2022 Jan 5;12:808285. doi: 10.3389/fmicb.2021.808285 (PMC8767002; doi:10.3389/fmicb.2021.808285)
Supplement: Supplementary file 13 [file Data_Sheet_1.DOCX]

**Exploring** **sexual dimorphism in the intestinal microbiota of the yellow drum (*Nibea albiflora*, Sciaenidae)**

**Haidong Li^1^, Lei Lu^1,2^, Ruiyi Chen^2^, Shanshan Li^1^, Dongdong Xu^2^^[[1]](#footnote-1)^***

^1^School of Fishery, Zhejiang Ocean University, Zhoushan 316022, China

^2^Key Lab of Mariculture and Enhancement of Zhejiang province, Zhejiang Marine Fisheries Research Institute, Zhoushan 316021, China

[Supplementary Figures 2](#_Toc89544353)

[Figure1 Design of breeding experiment. 3](#_Toc89544354)

[Figure 2 The rarefaction curves in all samples. 4](#_Toc89544355)

[Figure 3 Non-metric multi-dimensional scaling analysis (NMDS) showed the structure differences of the intestinal microbiota among the female, male and all-female based on Jaccard distance (A); NMDS analysis showed the structure differences of the intestinal microbiota among all groups based on Jaccard distance (B). 5](#_Toc89544356)

[Figure 4 The relative abundance of genera in all groups. 6](#_Toc89544357)

[Figure 5 Relationships between α-diversity metrics and growth indices. 7](#_Toc89544358)

[Figure 6 The distribution of topological roles of overall OTUs in the six groups. 8](#_Toc89544359)

# Supplementary Figures


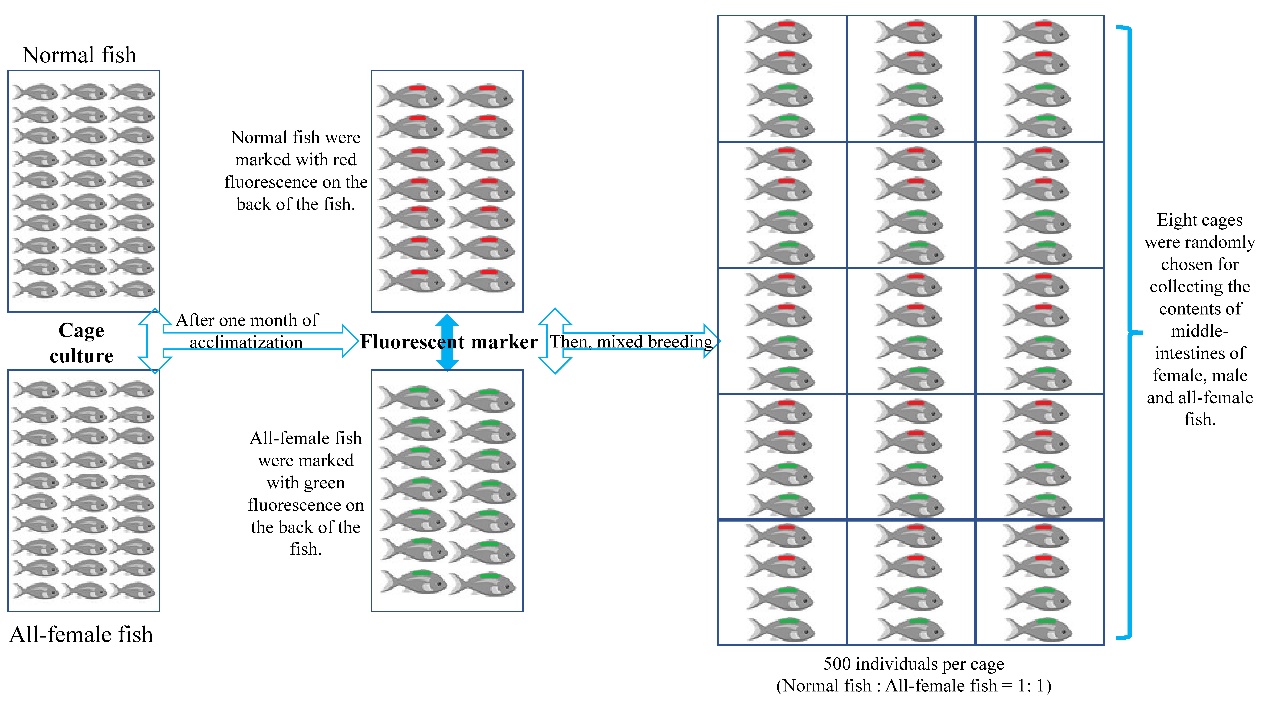


## Figure1 Design of breeding experiment.


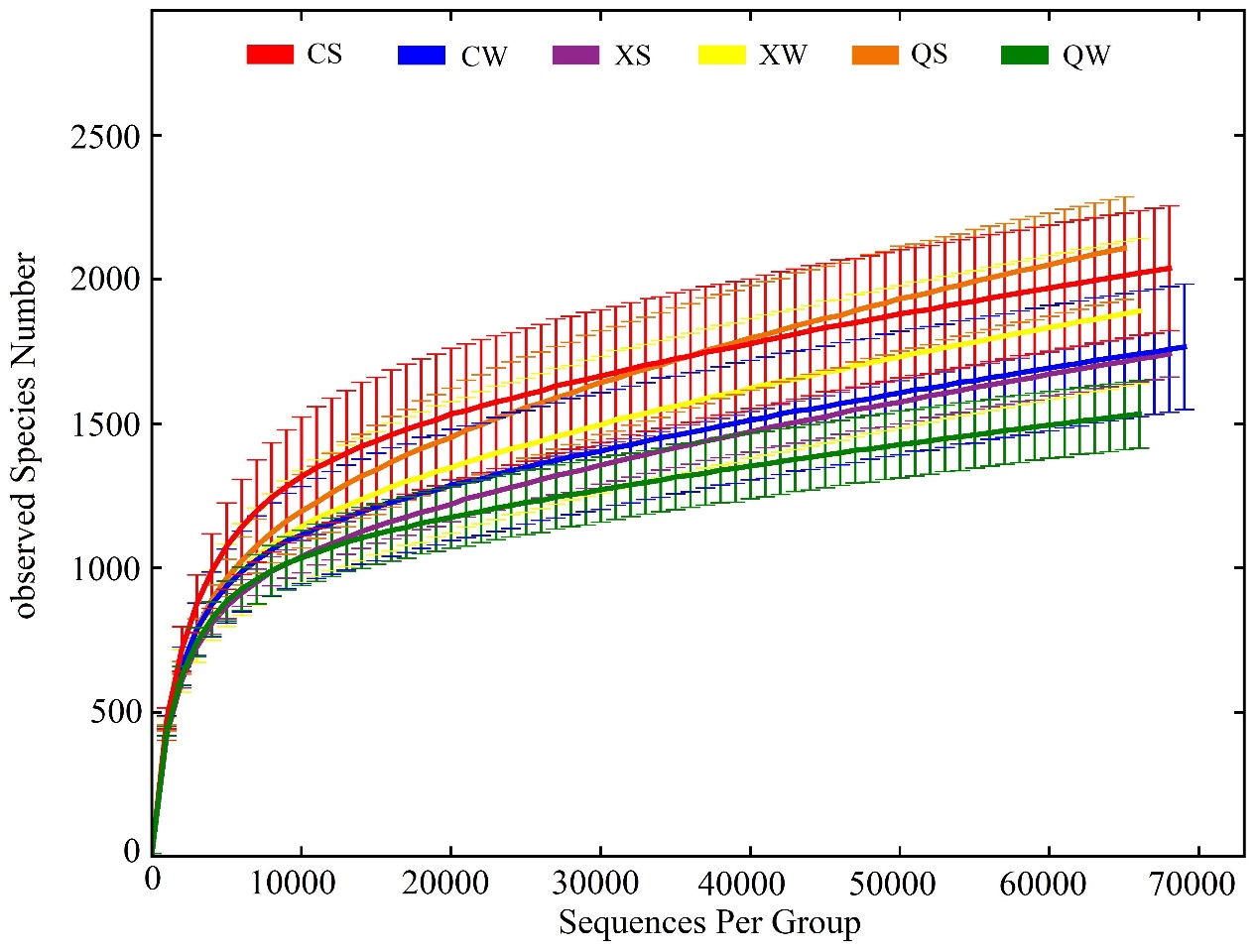


## Figure 2 The rarefaction curves in all samples.

The X, C and Q are respectively represented male, female, all-female fish. Two seasons: summer (S) and winter (W).


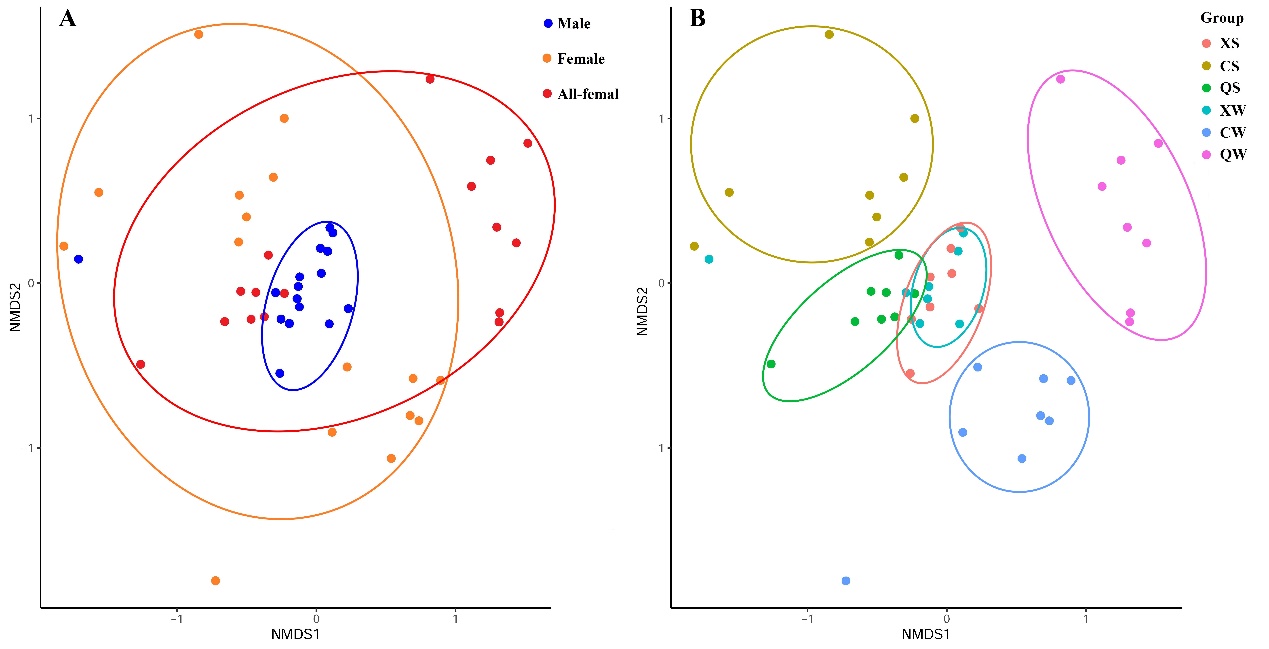


## Figure 3 Non-metric multi-dimensional scaling analysis (NMDS) showed the structure differences of the intestinal microbiota among the female, male and all-female based on Jaccard distance (A); NMDS analysis showed the structure differences of the intestinal microbiota among all groups based on Jaccard distance (B).

The X, C and Q are respectively represented male, female, all-female fish. Two seasons: summer (S) and winter (W).


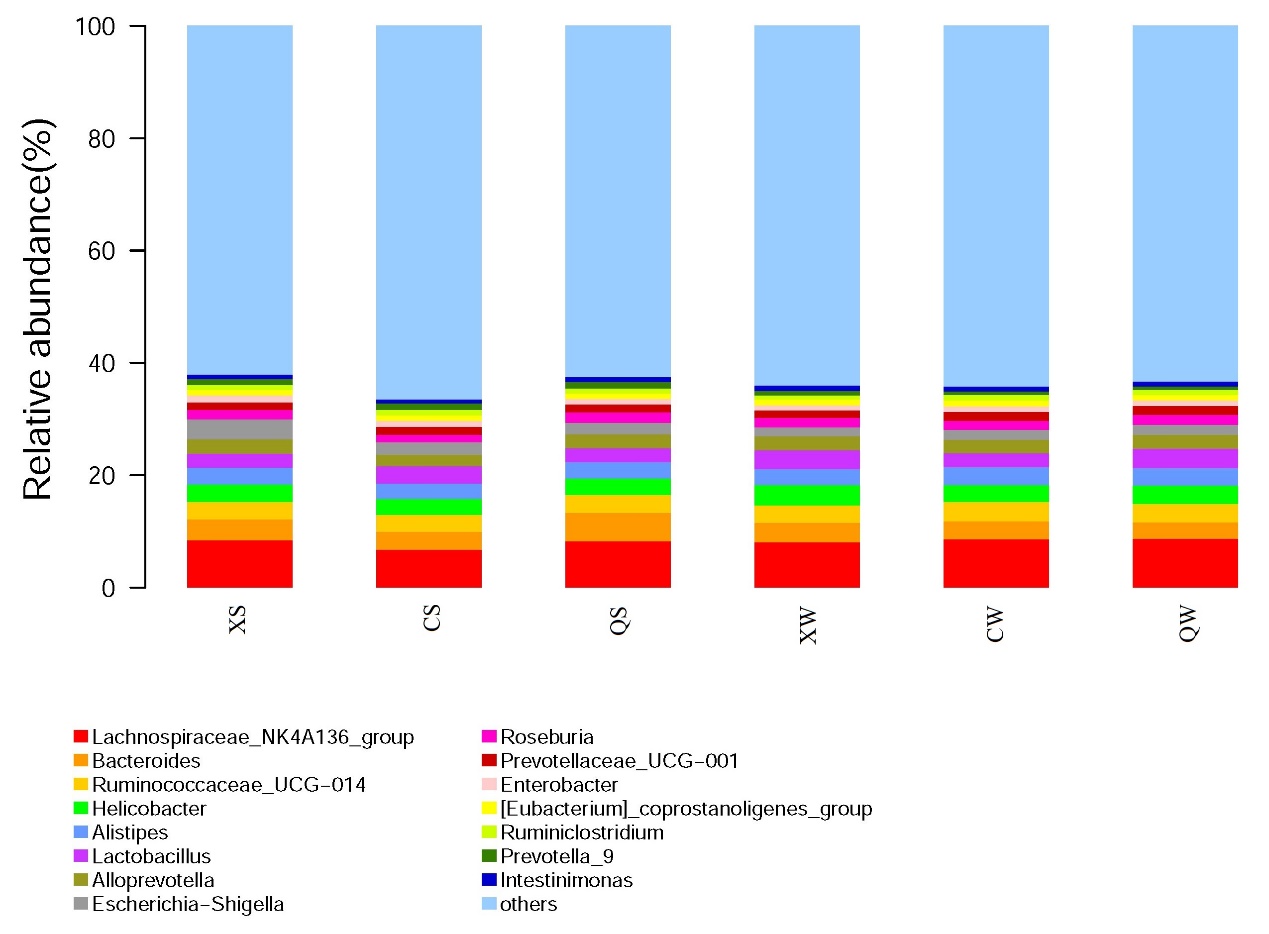


Figure 4 The relative abundance of genera in all groups.

The X, C and Q are respectively represented male, female, all-female fish. Two seasons: summer (S) and winter (W).


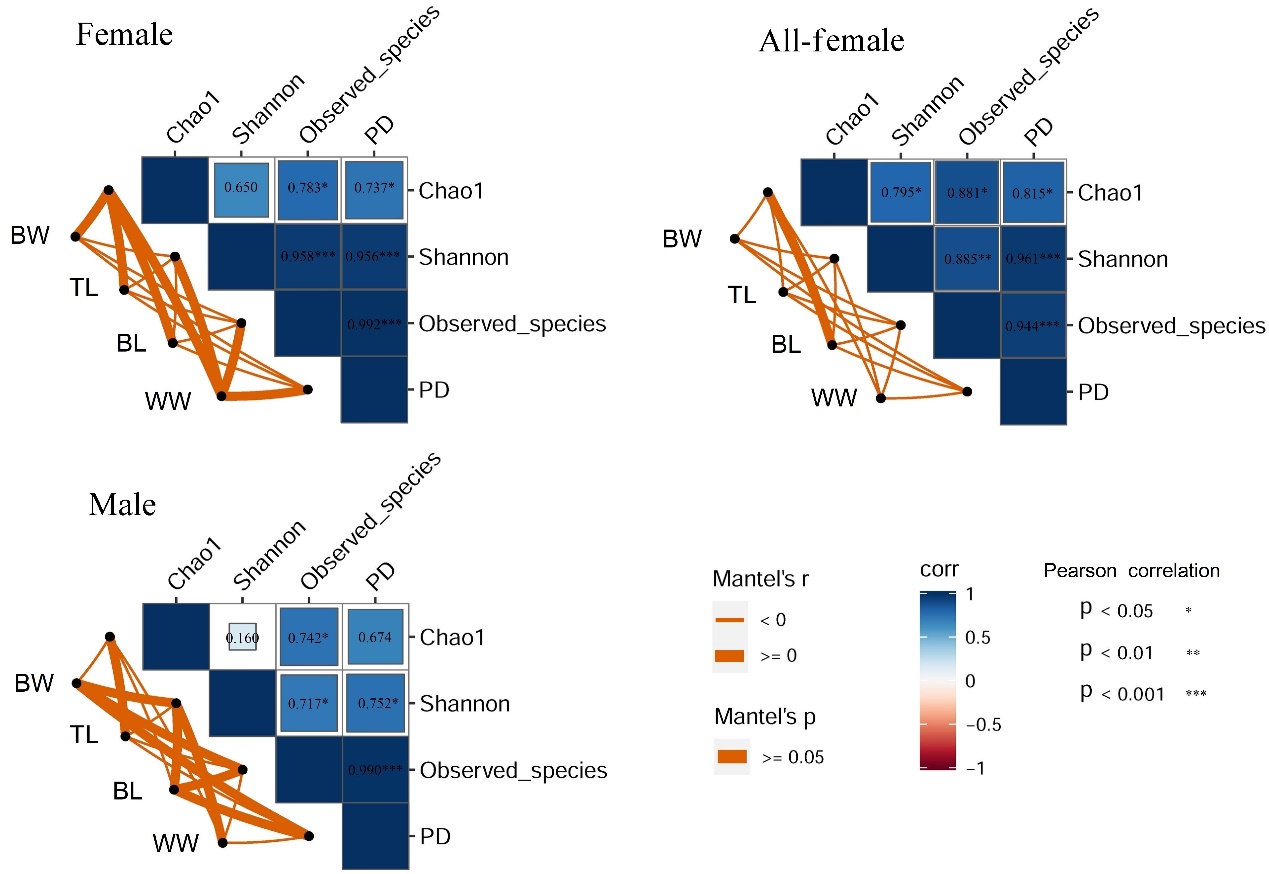


Figure 5 Relationships between α-diversity metrics and growth indices.

Growth indices: body length: BL; total length: TL; body weight: BW; whole viscera weight: WW. PD: phylogenetic diversity. Female: CS and CW groups; Male: XS and XW groups; All-female: QS and QW groups. Corr (correlation): red in the cell indicated a negative correlation, while bule in the cell indicated a positive correlation. Mantel’s r: r < 0 represented negative correlation; r > 0 represents positive correlation. The lines betweenα-diversity metrics and growth indices denoted correlations: red lines represented no significant correlation.


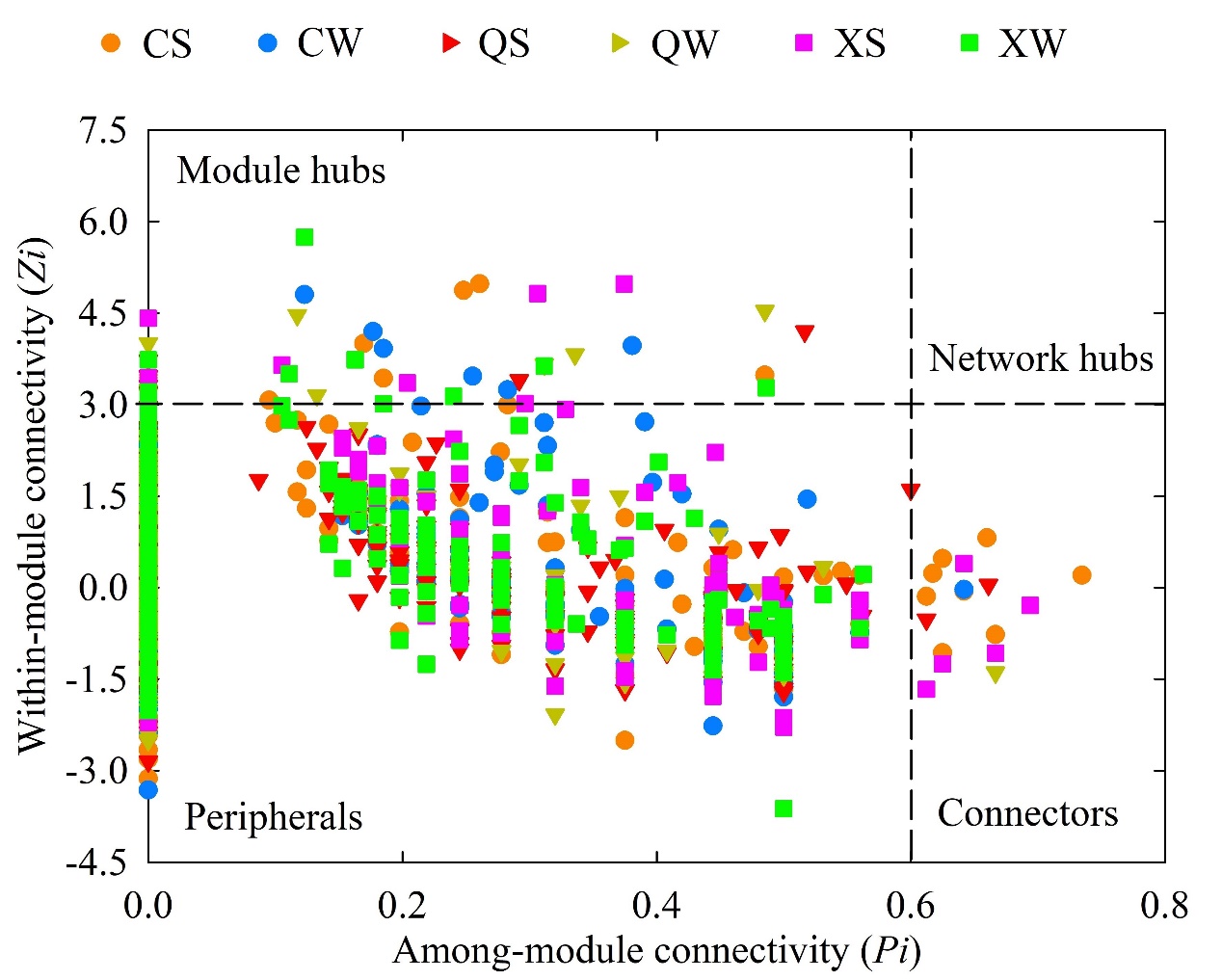


Figure 6 The distribution of topological roles of overall OTUs in the six groups.

Species with both a low Zi (value < 2.5) and a low Pi (value < 0.6) were peripheral species, i.e., they had only a few links, and these were almost always only to species within their module. Species with either a high value of Zi (≥ 2.5) or Pi (≥ 0.6) were module hubs, connectors and network hubs, as they were highly connected species linked to many other species within their own module. The X, C and Q are respectively represented male, female, all-female fish. Two seasons: summer (S) and winter (W).

1. * Corresponding author: Dongdong Xu; E-mail address: xudong0580@163.com [↑](#footnote-ref-1)
